# The Effect of Astaxanthin on Mitochondrial Dynamics in Rat Heart Mitochondria under ISO-Induced Injury

**DOI:** 10.3390/antiox12061247

**Published:** 2023-06-09

**Authors:** Roman Krestinin, Yulia Baburina, Irina Odinokova, Alexey Kruglov, Linda Sotnikova, Olga Krestinina

**Affiliations:** Institute of Theoretical and Experimental Biophysics, Russian Academy of Sciences, 142290 Pushchino, Moscow Region, Russia; rkrestinin@bk.ru (R.K.); byul@rambler.ru (Y.B.); odinokova@rambler.ru (I.O.); linda_sotnikova@mail.ru (L.S.)

**Keywords:** rat heart mitochondria, oxidative stress, fission, fusion, mytophagy, ISO-induced damage

## Abstract

Mitochondria are dynamic organelles that produce ATP in the cell and are sensitive to oxidative damage that impairs mitochondrial function in pathological conditions. Mitochondria are involved not only in a healthy heart but also in the development of heart disease. Therefore, attempts should be made to enhance the body’s defense response against oxidative stress with the help of various antioxidants in order to decrease mitochondrial damage and reduce mitochondrial dysfunction. Mitochondrial fission and fusion play an important role in the quality control and maintenance of mitochondria. The ketocarotenoid astaxanthin (AX) is an antioxidant able to maintain mitochondrial integrity and prevent oxidative stress. In the present study, we investigated the effect of the protective effect of AX on the functioning of rat heart mitochondria (RHM). Changes in the content of proteins responsible for mitochondrial dynamics, prohibitin 2 (PHB2) as a protein that performs the function of quality control of mitochondrial proteins and participates in the stabilization of mitophagy, and changes in the content of cardiolipin (CL) in rat heart mitochondria after isoproterenol (ISO)-induced damage were examined. AX improved the respiratory control index (RCI), enhanced mitochondrial fusion, and inhibited mitochondrial fission in RHM after ISO injury. Rat heart mitochondria (RHM) were more susceptible to Ca^2+^-induced mitochondrial permeability pore (mPTP) opening after ISO injection, while AX abolished the effect of ISO. AX is able to perform a protective function in mitochondria, improving their efficiency. Therefore, AX can be considered an important ingredient in the diet for the prevention of cardiovascular disease. Therefore, AX can be examined as an important component of the diet for the prevention of heart disease.

## 1. Introduction

One of the most common ailments in the world is considered to be heart damage, expressed in myocardial infarction with the subsequent occurrence of heart failure. Although mortality after myocardial infarction is declining, heart failure is increasing in prevalence and severity. Therefore, new therapies are needed to protect the myocardium in order to reduce the size of myocardial infarction, preserve the function of the left ventricular, and prevent heart damage [1]. In various pathologies, mitochondria are considered to be the organelles that are initially damaged. The involvement of mitochondria in the pathogenesis and progression of numerous diseases such as cancer, neurodegenerative and cardiovascular diseases, diabetes, traumatic brain injury, and inflammation is explained by the key role that the organelle plays in the consequences of events that culminate in cell death through various programmed and unprogrammed mechanisms of cell death [2].

Mitochondria are dynamic organelles that undergo fission and fusion. The processes are necessary for cell survival and adaptation to changing conditions, which are necessary for cell growth, division, and distribution of mitochondria [3]. When mitochondria are damaged, fusion relieves stress by allowing functional mitochondria to supplement dysfunctional mitochondria by diffusing and exchanging components between organelles. Fission is necessary to create new mitochondria. However, it also contributes to mitochondrial quality control by allowing the removal of damaged mitochondria [4,5]. In mammals, mitochondrial fission is mediated by dynamin-related protein1 (Drp1), which is a large GTPase. Drp1 is a cytosolic protein involved in the mitochondrial outer membrane and constricts the mitochondria, resulting in the separation of the mitochondria into two individual organelles [6,7]. Mitochondrial fusion is mediated by proteins such as mitofusin 1 (Mfn1) and 2 (Mfn2) and optic nerve atrophy 1 (OPA1) [8]. Mfn1 and Mfn2 are dynamin-coupled GTPases that are involved in the fusion of the outer mitochondrial membrane, whereas OPA1 is a dynamin-related GTPase that is involved in the fusion of the inner mitochondrial membranes [9]. Due to the action of drugs, mitochondrial functioning can improve while the number of healthy mitochondria increases. This may occur through the inhibition of excessive mitochondrial fission, which promotes mitochondrial fusion or removes damaged mitochondria. This may prove to be a promising approach to the treatment of various pathological conditions of the heart [10]. Moreover, there are drugs that regulate mitochondrial dynamics, leading to inhibition of Drp1, and reduce CVB3 (coxsackievirus B3, a pathogen of the cardiovascular system)-induced damage to the heart [11]. Fusion promotes the mixing of mitochondria, ensures the integrity and homogeneity of the mitochondrial network, protects against the loss of mitochondrial DNA (mtDNA), and maintains optimal bioenergetic activity. Fission promotes the spread and inheritance of mitochondria and aids in the isolation of dysfunctional mitochondria from the intact network and their selective removal by mitophagy [12].

It is known that there is a relationship between the change in the permeability of the mitochondrial membranes accompanied by the opening of the mitochondrial transition pore (mPTP) and fusion (e.g., cristae remodeling). Carbonyl cyanide m-chlorophenyl hydrazone (CCCP, ionopore), which dissipates the membrane potential and promotes mPTP opening, results in the inhibition of fusion and fragmentation of mitochondria [13]. Indeed, a drop in mitochondrial membrane potential due to mPTP opening is able to inhibit mitochondrial fusion [14].

Recently, we studied the influence of the AX on the functioning of RHM with ISO damage and found that the AX is able to protect mitochondria and may be preventing the development of heart disease [15,16]. In addition, we have identified a 30 kDa protein as prohibitin (PHB), which changes its content in heart mitochondria in rats with ISO damage [17]. There are two highly homologous subunits, PHB1 and PHB2 [18]. PHBs perform various functions in mitochondria; one of them is considered to be quality control of mitochondrial proteins [19]. In mammals, several mitophagy receptors have been characterized, among them PHB2. PHB2 is an internal mitochondrial protein that is critical for targeting mitochondria for autophagic degradation. In addition, the lack of PHB2 causes a defect in mitochondrial clearance [20].

In this work, we studied the effect of AX on changes in the heart tissue, the functional state of mitochondria, and the content of proteins responsible for mitochondrial dynamics in the heart mitochondria of rats with damage initiated by isoproterenol (ISO).

## 2. Materials and Methods

### 2.1. Animals and Treatment

In the experiment, 16 male Wistar rats weighing 240–250 g (age 2 months) were used. The experiments were carried out in accordance with the regulations on conducting research on experimental animals (Order of the Ministry of Health of the Russian Federation of 12 August 1997 No. 755). The protocol was approved by the Commission on Biological Safety and Ethics of the Institute for Theoretical and Experimental Biophysics of the Russian Academy of Sciences (protocol No. 05/2022 dated 5 March 2022). There are four groups (four rats in each group). Group 1 of rats was the control group. AX (150 mg/kg, Natural, China, 150 mg/kg) was dissolved in olive oil and administered orally with 15 ga × 78 mm plastic feeding tubes (Instech, Plymouth Meeting, PA, USA) to group 2 rats for 2 weeks. Group 3 rats were subcutaneously injected with ISO (dissolved in water at 100 mg/kg) twice at 24 h intervals to induce ISO injury. Rats in group 4 were administered AX (150 mg/kg) for two weeks. In the last two days before slaughter, the rats were injected with ISO twice, with an interval of 24 h [21,22,23]. Rats in group 4 continued to receive AX during the ISO injection. Animals in groups 1 and 3 received olive oil in the quantities that animals in groups 2 and 4 received together with AX. RHM was isolated from each group of rats.

### 2.2. Histological Analysis

To obtain an objective picture of heart damage after ISO injection, identical regions of the left ventricle (LV) of the rat heart were taken. LV fragments for histological analysis were cut as quickly as possible from the whole heart immediately after removal from the rat chest, washed, and fixed in neutral buffered formalin (NBF) for 24 h according to the standard method at room temperature [24]. After fixation, the LV fragments were washed three times to remove excess phosphates in distilled water and immersed in Compound Tissue Tek (Sakura, Japan) for 12 h at +4 °C. A series of every three consecutive 9 μm thick LV transverse sections was obtained using a cryotome (Shandon CRYOTOME 620E, Thermo Fisher Sci., Waltham, MA, USA) in 30 μm increments. Each of the three adjacent sections was stained with hematoxylin and eosin (H&E) and two differential trichrome stains. To obtain a general picture of the degree of heart damage, survey histotopograms of preparations were obtained by gluing using a Nikon Eclipse Ti-E microscope station (Nikon, Japan) and Nis Elements AR4.13.05 (Build933) software (NIS-Elements software). To identify the tissue damage, subendocardial (mixed type of section of longitudinal and transverse myocardial muscle bundles) and subepicardial (mainly cross section of myocardial bundles) areas of the myocardium were separately evaluated and compared. Myocardial damage was assessed by staining in two ways: Masson’s trichrome stain(ing) and Lillie’s trichrome stain(ing) [25].

### 2.3. Isolation of Rat Heart Mitochondria

Rat heart mitochondria were isolated from the heart according to the standard method described in [23]. The heart was crushed and homogenized in a medium containing 75 mM sucrose (Sigma S7903, St. Louis, MO, USA), 10 mM Tris-HCl (pH 7.4), 225 mM mannitol (Sigma M4125, St. Louis, MO, USA), 0.5 mM EDTA (Sigma E9884, St. Louis, MO, USA), 0.5 mM EGTA (Sigma E3889, St. Louis, MO, USA), and 0.1% BSA (Sigma A6003, St. Louis, MO, USA). The homogenate was then centrifuged at 1000× *g* for 10 min. The resulting supernatant (with mitochondria) precipitated at 8500× *g* for 10 min. The precipitate containing mitochondria was washed with an isolation medium without EDTA or BSA at 8500× *g* for 10 min. The obtained pellet was suspended in the same medium. All procedures were carried out at 4 °C. The Bradford method was used to determine the protein concentration in mitochondria, which was 30–35 mg/mL.

### 2.4. Evaluation of the Mitochondrial Function

To determine mitochondrial functions, a multifunctional chamber was used where the resulting mitochondria (1 mg protein/mL) were incubated in a medium containing 125 mM KCl, 10 mM Tris (pH 7.4), and 2 mM K_2_HPO_4_ at 25 °C. Glutamate (5 mM) and malate (5 mM) were used as respiratory substrates. A multifunctional chamber with built-in tetraphenylphosphonium ion (TPP^+^) and Ca^2+^-selective electrodes (Nico-Analyt, Moscow, Russian Federation) was used. The rates of oxygen consumption were measured with the help of a Clarke-type electrode [26]. Respiratory activities were determined after adding 150 μM ADP and 1.5 μM olygomycin (Sigma 75351, Saint Louis, MO, USA) to RHM in a closed chamber. The respiratory control index (RCI) was calculated as a ratio of V_st.3_ to V_st.4_. The rates of oxygen consumption were estimated (V_st.2_, V_st.3_, and V_st.4_; ng-atom O min^−1^ mg^−1^ of protein). The Ca^2+^-induced dissipation of the membrane potential (ΔΨm) was determined as the rate of TPP^+^ influx (V^TPP+^_in_, nmol min^−1^ mg^−1^ of protein) since the change in this indicator reflects a drop in ΔΨm. The opening of mPTP was initiated by [Ca^2+^] at a threshold concentration, which was achieved by sequential addition of Ca^2+^, where each addition was 50 nmol per mg of protein. Mitochondrial swelling was measured by the change in light scattering in the mitochondrial suspension at 540 nm (A540) on a Tecan I-Control Infinite 200 spectrophotometer (Infinite 200 Tecan, Mannedorf, Switzerland) at 25 °C. The concentration of mitochondrial protein in the well was 0.5 mg protein/mL. Swelling was stimulated by 160 nmol Ca^2+^ per mg of protein, which corresponded to the threshold calcium concentration under control conditions. The standard incubation medium for the swelling of mitochondria contained 125 mM KCl, 10 mM Tris, 2 mM KH_2_PO_4_, 5 mM glutamate, and 5 mM malate.

### 2.5. Electrophoresis and Immunoblotting

The samples of mitochondrial lysates (1 mg/mL) dissolved in Laemmli buffer (Bio-Rad, Hercules, CA, USA) were added to each line at 20 μg and divided by electrophoresis (12.5% SDS-PAGE). The proteins were transferred from the gel onto a nitrocellulose membrane (0.2 µm). The membranes were blocked in Roti-block solution (Carl Roth GmbH + Co., Karlsruhe, Germany) for 1 h. The membranes were stained with the polyclonal antibodies to DRP1 (Elabscience, Houston, TX, USA) at dilution 1:2000, Mfn2 (Elabscience, Houston, TX, USA) at dilution 1:2000, OPA1 (Cloud-Clone Corp., Katy, TX, USA) at dilution 1:1000, PHB2 (Cusabio, Houston, TX, USA) at dilution 1:1000, and PINK1 (Cusabio, Houston, TX, USA) dilution 1:500 overnight. The polyclonal anti-Tom20 antibodies (Cell Signaling, Danvers, MA, USA) at dilution 1:1000 were used to normalize the protein. Immunoreactivity was determined using secondary antibodies conjugated with horseradish peroxidase (Jackson Immuno Research, West Grove, PA, USA). Peroxidase activity was determined with an ECL (Bio-Rad, Hercules, CA, USA) using a ChemiDoc Touch imaging system (Bio-Rad, Hercules, CA, USA). Protein bands were quantified by densitometry (Image Lab software, Bio-Rad, Hercules, CA, USA) as the ratio of proteins to Tom20.

### 2.6. Statistical Analysis

For statistical analysis, we took the average value of the parameters calculated from the results of four to six experiments ± SD. The statistical significance between the pairs of mean values was evaluated using the Student–Newman–Keul (in the Supplementary File, the four data points in the graph were added). The difference was considered significant at *p* < 0.05.

## 3. Results

**Figure 1:**
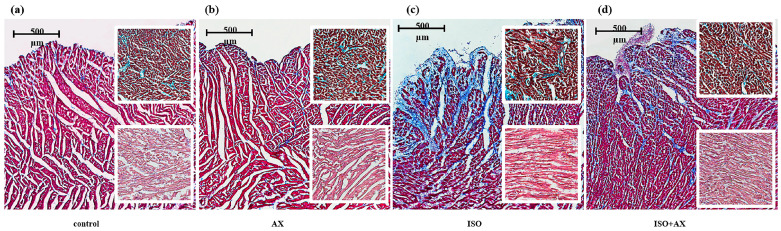
Transmural histotopograms of left ventricular (LV) tissue from rat hearts. (**a**)—the tissue samples from control group 1; (**b**)—the tissue samples from rats after AX administration, group 2; (**c**)—the tissue samples from rats with ISO injection, group 3; (**d**) the tissue samples from rats after AX administration and injected with ISO, group 4. Main images reflect Masson’s trichrome stain (collagen/fibrosis—blue, muscles and other tissues—red, cell nuclei—brown), ruler 500 μm. Upper inserts—enlarged fragments of the subendocardial zone of the myocardium with predominantly cross sections of myocardial fibers; Lilly’s trichrome stain (collagen/fibrosis shown in blue, muscles and other tissues in red-brown, cell nuclei in brown-black), ruler 100 μm. Lower inserts—enlarged fragments of the middle zone of the myocardium; H&E (cell nuclei in blue, erythrocytes in red, muscle tissue in pink); ruler 100 μm.

At first, we compared changes in heart tissue under our experimental conditions (Figure 1). To detect myocardial damage and its localization, transmural histotopograms of left ventricular samples from all groups were evaluated. In addition, we compared changes in the subepicardial (Figure 1, upper insets in figures), midline (Figure 1, middle insets), and subendocardial (Figure 1, lower insets) zones of the myocardium. When comparing samples of groups 1 (a) and 2 (b), no differences were found in all studied areas of the myocardium. The histological parameters of groups 1 and 2 corresponded to the standards of the structure and architectonics of the myocardium of this age group in Wistar rats. For heart fragments from groups 3 (c) and 4 (d), subendocardial myocardial involvement was observed. However, myocardial damage in group 4 was less pronounced compared to group 3. In addition, in the samples of group 4, the subepicardial and median sections of the heart wall were practically unaffected. On the contrary, in the samples of group 3, there were signs of fibrosis-like changes in the middle region of the myocardium, as well as areas of fusion of edematous muscle fibers and the appearance of subsegmental contractures (compare the upper and lower inserts in Figure 1c,d). In addition, the content of alanine aminotransferase, aspartate aminotransferase, lactate dehydrogenase, troponin I and myoglobin decreased in tissue lysates of the left ventricle of the rat heart (Supplementary File, Figure S1) under ISO-induced injury. Despite the fact that ISO causes heart failure, in our study we talk about ISO-induced damage to the heart since ECHO of the heart is not possible to study.

Then, we studied the effect of AX on the functional state of heart mitochondria in rats with ISO-induced injury. The injection of ISO (subcutaneously) to induce heart damage is a model accepted in the scientific community [21,22,23].

**Figure 2:**
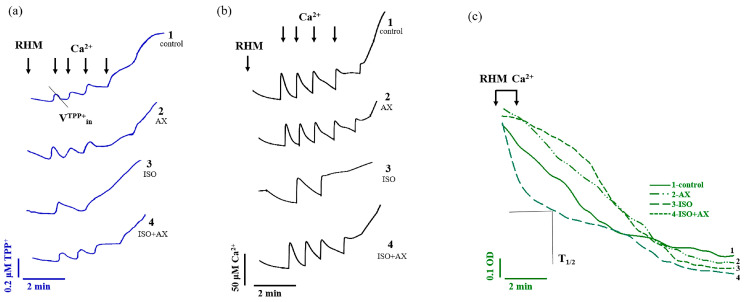
The effect of AX and ISO on Ca^2+^ transport in rat heart mitochondria upon Ca^2+^-induced mPTP opening. (**a**)—Curves reflecting the distribution of the TPP^+^ ion during Ca^2+^-induced mPTP opening; (**b**)—Curves reflecting Ca^2+^ fluxes; (**c**)—Curves of swelling of RHM.

Here, we measured respiratory activities under our experimental conditions (Table 1). The rate of oxygen consumption in state 2 (V_st.2_) reflects the substrate-dependent respiration of isolated mitochondria, and state 3 (V_st.3_) is the rate of oxygen consumption by mitochondria in the phosphorylating state. The administration of AX (group 2) did not affect the rate of oxygen consumption in V_st.2_ and V_st.3_ compared with the control. In RHM isolated from rats after ISO injection, V_st.2_ and V_st.3_ decreased by 20% and 30%, respectively, compared to the control. V_st.2_ and V_st.3_ in RHM isolated from group 4 (AX + ISO) increased by 36 and 33%, respectively, relative to RHM isolated from the group of rats after ISO injection (group 3). The rate of oxygen consumption in state 4 (V_st.4_, the rate of respiration in the adjusted state after exhaustion of excess ADP) in RHM after injection of ISO increased by 40% compared with the control. After the administration of AX in combination with ISO, V_st.4_ in RHM did not change compared with the control but decreased by 34% relative to V_st.4_ in RHM isolated from ISO-treated rats. An indicator of mitochondrial performance is the respiratory control index (RCI), which is calculated as the ratio V_st.3_/V_st.4_. It should be noted that RCI in rats that were administered with AX and rats that received AX followed by an injection of ISO did not differ from the control. RCI in the heart mitochondria of rats after ISO injection decreased approximately 2-fold compared to the control, while RCI in RHM treated with AX followed by ISO injection increased 2-fold compared to RCI in RHM injected with ISO. It is believed that oxidative stress affects the functional state of mitochondria and causes mitochondrial dysfunction [27]. Here, we investigated the effect of AX in the case of ISO-induced heart injury on the CRC, membrane potential (ΔΨm), and mitochondrial swelling in RHM isolated from every group of rats (Figure 2).

Figure 2a reflects the curves of changes in Ca^2+^ flow in RHM. Each supplement was added to the mitochondria to reach a threshold Ca^2+^ concentration for the mPTP to open at 50 nmol per mg of protein. In RHM isolated from groups 1 (control) and 2 (AX administration), the fifth Ca^2+^ pulse, in RHM isolated from group 3 (ISO injection), the second addition of Ca^2+^, and the fourth Ca^2+^ pulse in RHM isolated from group 4 (AX administration + ISO injection) led to the opening of the mPTP. Similar results were obtained for the change in the rate of TPP^+^ influx during Ca^2+^-induced pore opening (Figure 2b). Since one of the reasons for the change in mitochondrial membrane permeability is believed to be mitochondrial swelling [28], we measured the mitochondrial swelling for every group of rats. Figure 2c shows the curves of Ca^2+^-induced mitochondrial swelling for every investigated group. An amount of 160 nmol Ca^2+^ per mg of protein was required to initiate mitochondrial swelling. This Ca^2+^ addition corresponded to the threshold Ca^2+^ concentration in RHM from group 1.

**Figure 3:**
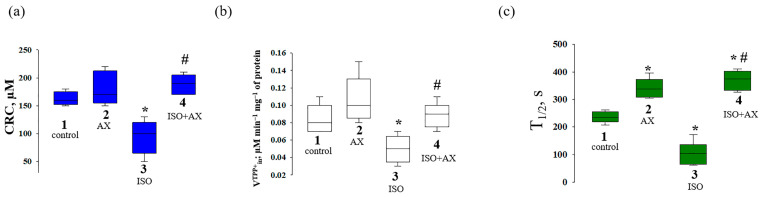
Quantitative analysis of the rate of TPP^+^ influxes, Ca^2+^ retention capacity, and mitochondrial swelling in rat heart mitochondria isolated from the rats of every group. (**a**)—CRC reflects the threshold Ca^2+^ concentration required to open the mPTP; (**b**)—the rate of TPP^+^ influx reflecting Ca^2+^-induced drop ΔΨm (V^TPP+^_in_, nmol min^−1^ mg^−1^ of protein); (**c**)—time to reach half-maximal light scattering (T_1/2_) reflecting the rate of swelling of mitochondria. The data are presented as the means ± SDs of five independent experiments. * *p* < 0.05 a significant difference compared with control (group 1); *# p* < 0.05 a significant difference relative to ISO (group 3).

The quantitative characteristics of the CRC, the rate of TPP^+^ influx, and the swelling of mitochondria are indicated in Figure 3. The chronic administration of AX and the combined effect of AX and ISO did not change the CRC in RHM compared to the control (Figure 3a). However, in the RHM from ISO-injected rats, CRC decreased by approximately 40% compared to the control. The joint action of AX and ISO increased the CRC in RHM by two times compared to the effect of ISO alone. AX did not change the rate of TPP^+^ influx into mitochondria compared to that in RHM from the control group, whereas ISO reduced the rate of TPP^+^ influx by 41% compared to the control. The influence of AX with ISO increased the rate of TPP^+^ influx by 70% relative to that in RHM from ISO-treated rats and did not change the rate of TPP^+^_in_ in the control rats.

The average half-maximal (T_1/2_) value of mitochondrial Ca^2+^-induced swelling is shown in Figure 3c. Mitochondrial swelling is measured by the time to reach the half maximum of the light scattering signal, which reflects the rate of mitochondrial swelling. The half-maximal value of mitochondrial swelling in rats from group 2 increased by 13%, indicating a slowdown in the rate of swelling, while ISO decreased T_1/2_ by 40% relative to the control, and the rate of swelling accelerated. The administration of AX in combination with ISO increased the T_1/2_ by 11% in comparison with the control and by 80% relative to ISO alone. ISO impairs mitochondrial membrane permeability and makes mitochondria more sensitive to Ca^2+^, while AX abolishes this effect.

**Figure 4:**
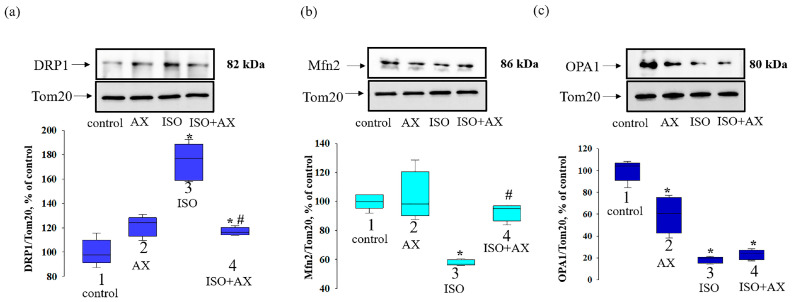
The influence of AX and ISO change of DRP1, Mfn2, and OPA1 in RHM. (**a**) upper part—Western blot with antibody to DRP1. Tom20 was used for normalization of proteins. Lower part—quantitative characteristic presented as the ratio of DRP1 to Tom20; (**b**) upper part—Western blot with antibody to Mfn2, lower part—quantitative characteristic presented as the ratio of Mfn2 to Tom20; (**c**) upper part—Western blot with antibody to OPA1, lower part—quantitative characteristic presented as the ratio of OPA1 to Tom20. The data are presented as the means ± SDs of four independent experiments. * *p* < 0.05 significant values compared with control (group 1); # *p* < 0.05 significant values relative ISO to (group 3).

Mammalian mitochondria are thought to form a highly dynamic reticular network in which fusion and fission events constantly occur, with the relative extent of these two events affecting mitochondrial quality control [29]. Therefore, we investigated the change in the content of proteins involved in fission (DRP1) and fusion (Mfn2, OPA1) in RHM under our experimental conditions (Figure 4). The upper part of Figure 4 shows Western blots stained with antibodies to DRP1 (a), Mfn2 (b), and OPA1 (c). The lower part of the figure indicates the quantitative analysis of protein bands normalized to Tom20. We observed that the content of DRP1 (Figure 4a) increased by 75% in the RHM from ISO-injected rats compared to the control (bar 3 vs. 1). AX administration did not change the level of DRP1 (bar 2 vs. 1), while the combination effect of AX and ISO enhanced DRP1 content by 17% relative to the control (bar 4 vs. 1) and decreased by 67% compared with ISO alone (bar 4 vs. 3).

AX administration did not affect the level of Mfn2 (Figure 4b) but decreased OPA1 content (Figure 4c) by 40% compared with the control (bar 2 vs. 1). The ISO decreased the content of Mfn2 and OPA1 by 43% and 80%, respectively, relative to the control (bar 3 vs. 1). The effect of AX with ISO did not change the content of Mfn2, but the OPA1 content was decreased by 77% compared with the control (bar 4 vs. 1), while compared to ISO alone, Mfn2 levels increased by 60% (bar 4 vs. 3).

PHBs play a role in OPA1-dependent mitochondrial fusion [30]. Moreover, suppression of PHB2 leads to the inhibition of mitophagy [31]. Therefore, at the next stage of our study, we checked the change in the levels of PHB2, PINK1, and CL under our experimental conditions.

**Figure 5:**
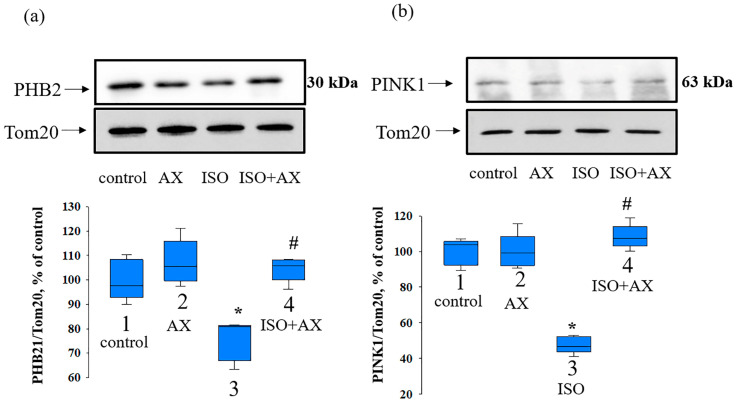
The influence of AX and ISO change of PHB2 and PINK1 in RHM. (**a**) upper part—Western blot with antibody to PHB2. Tom20 was used for normalization of protein. Lower part—quantitative characteristic presented as the ratio of PHB2 to Tom20; (**b**) upper part—Western blot with antibody to PINK1, lower part—quantitative characteristic presented as the ratio of PINK1 to Tom20. The data are presented as the means ± SDs of four independent experiments. * *p* < 0.05 significant values compared with control (group 1); # *p* < 0.05 significant values relative to ISO (group 3).

Figure 5 demonstrates the influence of AX on the change in PHB2 (a) and PINK1 (b) content in the heart mitochondria isolated from every group of rats. AX did not significantly affect the content of PHB2 and PINK1 (bar 2 vs. 1), whereas ISO decreased the level of PHB2 and PINK1 by 30 and 50%, respectively, relative to the control (bar 3 vs. 1). The combined effect of AX with ISO resulted in a 40% increase in PHB2 and a 50% increase in PINK1 content compared to ISO alone (bar 4 vs. 3), but did not change compared to the control (bar 4 vs. 1).

## 4. Discussion

It is generally accepted that mitochondrial fission and fusion play an important role in quality control and the preservation of mitochondria. To maintain the correct morphology necessary for normal functioning, mitochondria undergo processes of fission and fusion. The fission and fusion of mitochondria play a crucial role in maintaining the functional state of mitochondria when cells experience various types of stress [32]. The process of mitochondrial fusion allows the transfer of soluble and membrane components between mitochondria [33]. Fusion can be thought of as a complementation mechanism by which damaged or poorly functioning mitochondria can maintain their function. Dysfunctional mitochondria can lose their ability to fuse by inactivating fusion or activating fission mechanisms to prevent damaged mitochondria from being re-incorporated into the healthy mitochondrial network [32]. Under stress, the transmembrane potential decreases, and the frequency of mitochondrial fission exceeds the frequency of their fusion. This is a protective mechanism to prevent damage to the mitochondrial network as a whole [34].

The ketocarotenoid astaxanthin, present mainly in marine organisms, is attractive for study due to its diverse biological and physiological properties. Numerous researchers suggest that AX may maintain and protect mitochondrial ETC integrity and oxidative phosphorylation from oxidative stress [35]. In our study, the results of the histological analysis suggest that the use of AX reduces the degeneration and postischemic edema of the cardiac muscle fibers, as well as the myocardial damage induced by ISO. These results indicate a protective effect of AX. In addition, we observed that AX improved the functional state of heart mitochondria in rats after ISO injection. Moreover, RHM from ISO-injected rats was more susceptible to mPTP induction, CRC decreased, and mitochondrial swelling accelerated, while AX abolished the effects of ISO, wherein CRC increased and the rate of mitochondrial swelling decreased.

The mitochondrial dynamics are regulated by changes in expression between proteins responsible for fission and fusion. When mitochondria coalesce, the outer membranes fuse first, which is mediated by Mfn1/Mfn2, and then, with the help of OPA1, the inner membranes fuse [36,37]. A drop in membrane potential initiates the opening of mPTP and inhibits mitochondrial fusion [14]. Moreover, mitochondrial fusion was previously reported to occur preferentially between mitochondria with higher ΔΨm and generate an isolated subpopulation of non-fused mitochondria. Moreover, a physiologically significant decrease in ΔΨm has been shown to affect the prevalence of fusion. Each fusing mitochondria requires an intact ΔΨm to achieve matrix fusion, while damaged mitochondria with altered ΔΨm do not participate in the fusion process [38]. In our studies, we noticed that ISO reduced the content of Mfn2 and OPA1, while the level of DRP1 increased. Ong and coauthors reported that inhibition of Drp1 reduced cell death in isolated mouse cardiomyocytes in an ischemia/reperfusion injury model and reduced the size of MI in the heart of mice subjected to acute myocardial ischemia-reperfusion [39]. In addition, it has been shown that an increase in mitochondrial fission and a decrease in mitochondrial fusion are observed during cardiac reperfusion [40]. Researchers have shown that levels of mitochondrial fusion proteins decrease in cardiac hypertrophy and heart failure. Another study demonstrated that Mfn2 and OPA1 levels are reduced, while DRP1 and FIS1 levels are elevated in heart failure in dogs and humans [41]. AX is able to enhance mitochondrial fusion and inhibit mitochondrial fission in APP/PS1 transgenic mice [42]. On the contrary, under the combined effect of AX and ISO, the expression of Mfn2 increased; however, OPA1 did not change, while DRP1 decreased. Probably, under our experimental conditions, the process of fusion of the outer membranes but not the inner ones took place.

PHB2 is a ubiquitous protein localized in the mitochondrial inner membrane that plays a key role in the homeostasis of cellular energy metabolism [43]. Moreover, PHB2 is critical for mitochondrial structural and functional integrity, modulation of mitochondrial dynamics, mitophagy, and mitochondrial quality control [20,31]. PHB2 deficiency causes heart failure, which is fatal in mice [44]. In rats of the Sprague–Dawley line, the level of PHB decreased in the hypertrophy of the heart caused by ISO [45]. In our results, ISO led to an increase in degeneration and postischemic edema of the heart muscle fibers, while the level of PHB2 decreased. In addition, loss of PHB2 has been shown to affect mitochondrial morphology in C. elegans, where PHB2 depletion resulted in mitochondrial fragmentation and disorganization [46], while in mouse embryonic fibroblasts (MEF), the absence of PHB2 resulted in increased mitochondrial fission and the deprivation of mitochondrial cristae [47]. It should be noted that PHB2 acts as a receptor for the mitophagic mechanism [20] and is able to stabilize PINK1 in the mitochondria [31]. According to the results obtained, we observed that a decrease in PHB2 during ISO-induced damage led to a decrease in RINK1 in RHM. A decrease in the content of RINK1 implies a failure in the removal of accumulated damaged mitochondria. AX administration enhanced PHB2 expression, which, in turn, increased the content of PINK1 in RHM isolated from ISO-treated rats.

## 5. Conclusions

**Figure 6:**
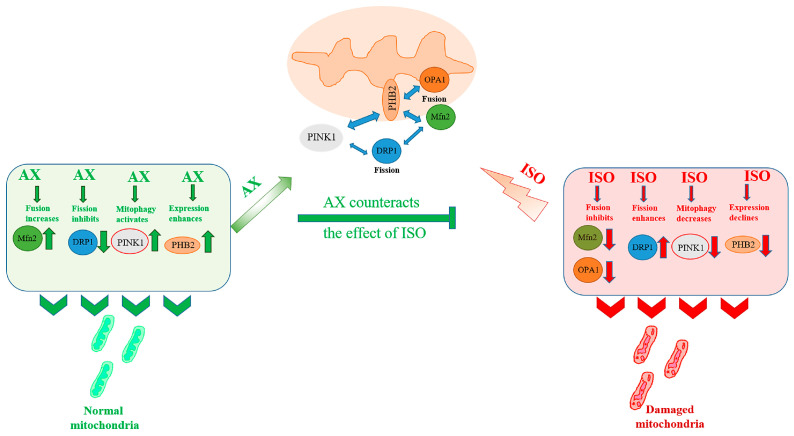
Hypothetical scheme of the influence of AX on ISO—induced damage to the functional state of rat heart mitochondria.

In the present study, we studied the effect of AX on the functional state, the change in mitochondrial fusion/fission, and the mytophagy of heart mitochondria from rats after ISO-induced damage. Histological analysis showed that AST reduced degeneration and edema of cardiac muscle fibers in ISO-induced injury. Figure 6 shows the proposed pattern of the AX effect on mitochondrial dynamics in heart mitochondria isolated from rats after ISO injection. ISO enhances mitochondrial fission and inhibits the fusion of both the outer membrane and the inner mitochondria. Under these conditions, mitophagy is reduced. These events probably occur due to the fact that the content of PHB2 in mitochondria decreases.

Damaged mitochondria that are not removed by mitophagy accumulate in the mitochondrial network, and mitochondrial functions are impaired (respiratory control index is decreased, mitochondria are more sensitive to opening mPTP, and mitochondrial swelling is accelerated). AX increases the content of PHB2, which in turn reduces fission, enhances the fusion of the outer mitochondrial membrane, and restores mitophagy. At the same time, the functional state of mitochondria improves (respiratory control of mitochondria and Ca^2+^ capacity increase, which leads to a slowdown in the opening of mPTP and inhibition of mitochondrial swelling). The use of AX in therapy may be a promising approach for the prevention and treatment of cardiovascular diseases.

## Figures and Tables

**Table 1:** The influence of AX and ISO on the respiratory activities of RHM isolated from every group of rats.

Parameters	Group №			
	1 (Control)	2 (AX)	3 (ISO)	4 (ISO + AX)
V_st2_, ng-atom O min^−1^ mg^−1^ of protein	7.25 ± 0.46	8.33 ± 0.47	4.52 ± 0.28 *	7.11 ± 0.32 ^#^
V_st3_, ng-atom O min^−1^ mg^−1^ of protein	43.70 ± 2.28	48.71 ± 4.11	34.36 ± 4.01 *	40.98 ± 2.75 ^#^
V_st4_, ng-atom O min^−1^ mg^−1^ of protein	7.48 ± 0.31	9.56 ± 0.37	12.58 ± 0.83 *	8.51 ± 0.86 ^#^
Respiratory control index	5.84 ± 0.88	5.10 ± 0.51	2.73 ± 0.21 *	4.81 ± 0.51 ^#^

The indicators of respiratory activity in states 2 (Vst.2), 3 (Vst.3), and 4 (Vst.4) were determined as the number of ng-oxygen atoms consumed by mitochondria per minute per mg of protein. RCI was calculated as a ration of Vst.3 to Vst.4. * *p* < 0.05, a significant difference compared to the control (group 1); ^#^ *p* < 0.05, a significant difference relative to RHM isolated from the group of rats that were injected with ISO (group 3).

## Data Availability

The data presented in this study are contained within this article and online in Supplementary Materials.

## Supplementary Materials

The following supporting information can be downloaded at: https://www.mdpi.com/article/10.3390/antiox12061247/s1. Figure S1. Histological analysis of the left ventricle of the heart of rats after AX administration and ISO injection. Figure S2. Influence of AX and ISO on changes in the content of myoglobin, troponin I, and LDH in rat heart tissue lysates.
