# Supplementary material for: The Effect of Astaxanthin on Mitochondrial Dynamics in Rat Heart Mitochondria under ISO-Induced Injury
Source: Antioxidants (Basel). 2023 Jun 9;12(6):1247. doi: 10.3390/antiox12061247 (PMC10295417; doi:10.3390/antiox12061247)
Supplement: Supplementary file 1 [file antioxidants-12-01247-s001.zip › antioxidants-2355679-supplementary.pdf]

Influence of AST and ISO on changes in the content of myoglobin, troponin I and LDH in rat heart tissue lysates.

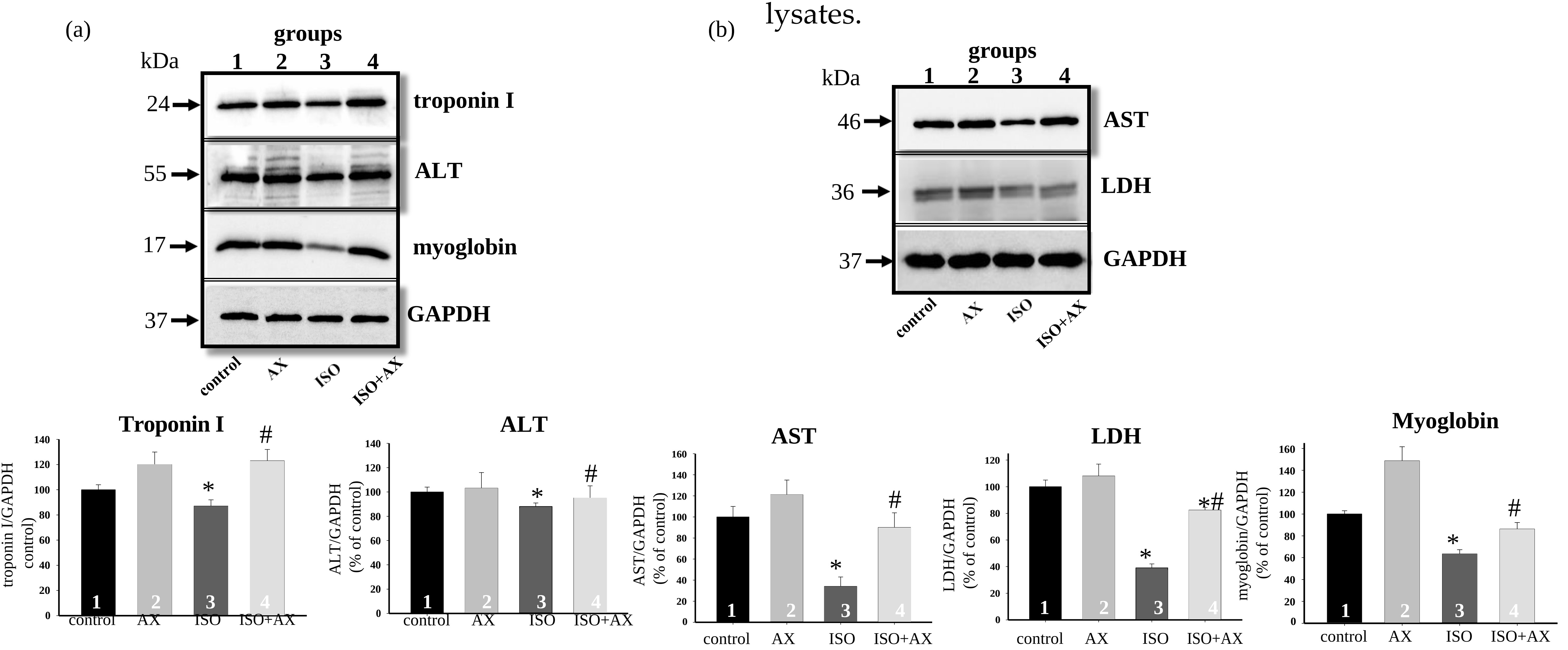

Figure S1. Protein samples were extracted and subjected to Western blot. GAPDH was used as a protein load control. (a) and (b) -immunostaining with antibodies to myoglobin, troponin I, LDH and GAPDH; GAPDH was used as a loading control.

In order to detect heart dysfunction in the presence of ISO, changes in the content of such proteins as myoglobin, troponin I, aspartate aminotransferase (AST), alanine aminotransferase (ALT), and lactate dehydrogenase (LDH) were checked. Heart tissue lysates were separated into proteins by electrophoresis in the Laemmli system, transferred to a nitrocellulose membrane, and stained with antibodies to the corresponding proteins. On figure 2 shows a Western blot of these proteins. As can be seen from the figure, the level of all proteins in tissue lysates of the heart from the group 3 (ISO injection) decreased. GAPDH was used as a load control for protein normalization. A decrease in the protein content in tissue lysates of rats after the injection of ISO (bar 3 vs. 1) indicates a violation of the function of the heart. AX abolished the effect of ISO and protein levels increased (bar 4 vs. 3).

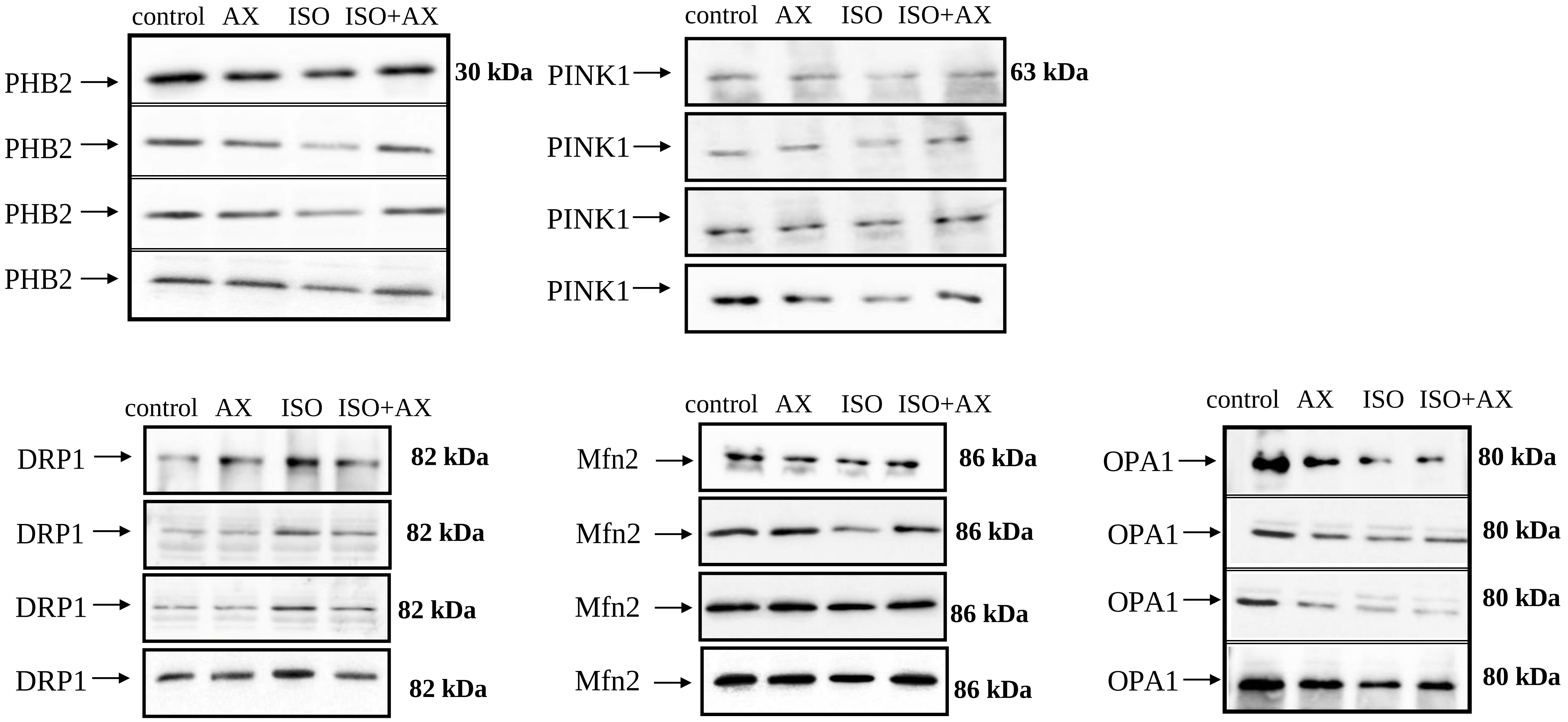

Figure S2. All WB used in the analysis of statistics in the article (Repeats)
